# Platelet-to-lymphocyte ratio at 24h after thrombolysis is a prognostic marker in acute ischemic stroke patients

**DOI:** 10.3389/fimmu.2022.1000626

**Published:** 2022-09-26

**Authors:** Ying-Ying Sun, Mei-Qi Wang, Yan Wang, Xin Sun, Yang Qu, Hong-Jing Zhu, Si-Ji Wang, Xiu-Li Yan, Hang Jin, Peng Zhang, Yi Yang, Zhen-Ni Guo

**Affiliations:** ^1^ Stroke Center, Department of Neurology, the First Hospital of Jilin University, Chang Chun, Jilin, China; ^2^ Jilin Provincial Key Laboratory of Cerebrovascular Disease, Chang Chun, China; ^3^ Neuroscience Research Center, the First Hospital of Jilin University, Chang Chun, Jilin, China

**Keywords:** platelet-to-lymphocyte ratio, acute ischemic stroke, intravenous thrombolysis, outcome, death

## Abstract

**Background:**

The changes in the platelet-to-lymphocyte ratio (PLR) before and after recombinant tissue plasminogen activator (rtPA) treatment and the time point at which the PLR is a potentially valuable prognostic predictor in patients wit ischemic stroke remain largely unknown. Therefore, the purpose of this study was to explore the characteristics of the PLR and evaluate their effects on clinical outcomes before and 24 h after rtPA treatment.

**Methods:**

This study included 741 consecutive patients with acute ischemic stroke who underwent intravenous thrombolysis with rtPA. We collected data on demographics, vascular risk factors, medication history, and other clinical information pertaining to all patients. Specifically, blood samples for PLR measurement were collected on admission and 24 h after stroke. The outcome was assessed by using the Modified Rankin Scale (mRS) at 3 months and whether death occurred within 3 months or not. Univariate and multivariate logistic regression analysis was used to assess the association of the PLR with the risks of poor outcome (mRS>2) and death. An individualized prediction model was established to predict poor outcome.

**Results:**

Of the 741 patients, 255 (34.4%) had poor outcome, and 43 (5.8%) died. The PLR significantly increased 24 h after rtPA in patients with poor outcome and death. Logistic analysis revealed that higher PLR 24 h after rtPA was independently associated with increased risks of poor outcome and death. However, the PLR on admission was not associated with the risks of poor outcome and death. The individualized prediction model for poor outcome based on the 24-h PLR exhibited favorable discrimination (areas under the curves of the training and validation groups: 0.743 and 0.729, respectively), calibration (*P* > 0.05), and clinical usefulness.

**Conclusions:**

We found the PLR to be a variable that potentially predicts the risks of poor outcome and death in patients with acute ischemic stroke 24 h after rtPA; however, it cannot make the same prediction on admission.

## Introduction

Acute ischemic stroke is one of the most common causes of disability and death worldwide, representing 80% of all stroke cases (1, 2). Although recombinant tissue plasminogen activator (rtPA) has been proved as the most effective treatment for acute ischemic stroke since the 1990s, limitations such as hemorrhagic transformation and narrow therapeutic window (4.5h) have posed challenges to the therapeutic qualities (3) (4, 5). Therefore, identifying the clinical indicators that potentially predict patient prognosis and determining possible means of controlling the risk factors at an earlier stage are of paramount importance.

Immune-inflammatory responses following ischemic stroke are associated with poor patient prognosis because of their role in exacerbating neuronal injury and damage to the blood-brain barrier (6). Emerging evidence suggests that platelets and lymphocytes play crucial roles in immune and inflammatory responses. Platelets are active participants in inflammatory reactions at sites of thrombosis. When a stroke occurs, platelets are activated rapidly and adhere to the injured endothelial surface. The inflammatory factors released by them can then further recruit inflammatory cells such as leukocytes to the site of injury and amplify the inflammatory response (7, 8). In contrast, lymphocytes are known to control inflammatory pathways by coordinating, healing, and repairing inflammation (9, 10). The platelet-to-lymphocyte ratio (PLR) is a bio-index combining platelets and lymphocytes, which can reflect thrombus formation and immune-inflammation pathways. The PLR has recently been reported as a potential novel biomarker in acute ischemic stroke intravenous thrombolysis treatment, playing an active role in the prediction of the functional outcomes (11, 12). However, these results were obtained from studies involving heterogeneous time points from symptom onset to the collection of blood samples for PLR calculation. The time point at which the PLR is collected is a potentially valuable predictor and the PLR changes before and after rtPA are largely unknown. Thus, this study aimed to (1) explore the changes in the PLR before and 24 h after rtPA and (2) evaluate their relationship with poor outcome and death in patients with acute ischemic stroke.

## Materials & methods

### Study population

**Figure 1:**
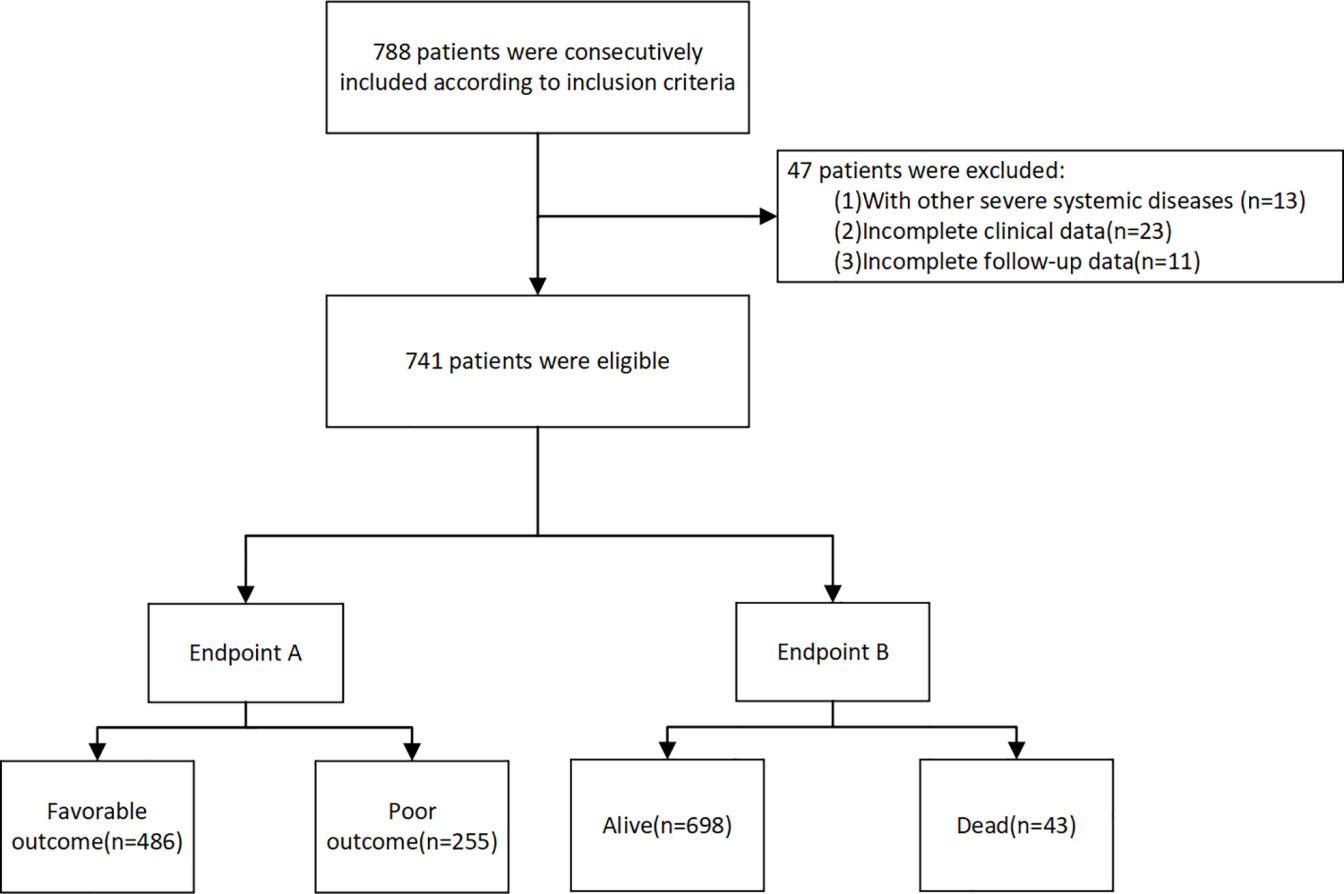
The flow chart of the study.

This retrospective study performed using prospectively collected data. Patients diagnosed with acute ischemic stroke who underwent intravenous thrombolysis with 0.9 mg/kg rtPA within 4.5h in our department, between April 2015 and December 2020, were performed. The exclusion criteria were as follows (1): with other severe systemic diseases such as cancer, rheumatism and hematological diseases (2); incomplete clinical data (3); incomplete follow-up data. The study flowchart is shown in **Figure 1**. This prospective observational study was registered (NCT05028868) and approved by the Ethics Committee of the First Hospital of Jilin University (2016–294). Written informed consent was signed by each participant or an appropriate agent.

### Data collection

Information on demographics (age and sex); vascular risk factors (smoking, alcohol consumption, hypertension, diabetes, coronary artery disease, atrial fibrillation and previous stroke); medication history (antihypertensive drugs, hypoglycemic, and antiplatelet agents); and other clinical information, including baseline systolic and diastolic blood pressure (SBP and DBP), baseline blood glucose, time to treatment, baseline National Institutes of Health Stroke Scale (NIHSS) score, infarction site (whether located in the anterior circulation), and the Trial of Org 10172 in Acute Stroke Treatment (TOAST) subtype were recorded. Blood samples were collected from all patients on admission and 24 h after thrombolysis for platelet- and lymphocyte-count assessment. The PLR was calculated as the ratio of the platelet count to lymphocyte count.

### Outcome

(A) Modified Rankin Scale (mRS): The mRS score was assessed at 3 months (13). A mRS score >2 denoted a poor outcome and that ≤2 a favorable outcome.

(B) Death was defined as a stroke related death within 3 months.

### Statistical analysis

IBM SPSS Statistics 23.0 and Stata 15.0 were used for statistical analysis. The Kolmogorov–Smirnov test was used to verify the normality of the distribution. All continuous variables were non-parametric distributions. Therefore, continuous and categorical variables were compared using the Mann–Whitney U-test and chi-squared test, which was then expressed in terms of median with interquartile range (IQR)and percentages, respectively. The paired Wilcoxon signed-rank test was performed to compare the PLR before and 24 h after rtPA. Univariate and multivariate logistic regression analysis was used to assess the association between PLR and outcome. Model 1 was unadjusted, Model 2 was adjusted for variables with P<0.05, Model 3 was adjusted for all other variables using enter method. An individualized prediction model for poor outcome was applied using multivariate logistic regression analysis and selected using a backward stepwise method with Akaike’s information criterion. Of the included cases, 70% and 30% were randomly assigned to training and validation groups, respectively. The nomogram was based on the data from the training group. The area under the receiver operating characteristics curve (AUC-ROC) was applied to assess model discrimination, and the calibration curve and Hosmer–Lemeshow test were applied to assess model fit in both the training and validation groups. Additionally, to further assess clinical usefulness, decision curve analysis (DCA) was performed by calculating the net benefit and plotting the net benefit against the threshold probability to derive a “decision curve”. DCA can determine the range of threshold probabilities that the model has value, and the magnitude of benefits (14). The model was validated by using a 10-fold cross-validation in all patients. P<0.05 was considered statistically different.

## Results

### Baseline characteristics of the patients

In our study, 788 rtPA-treated patients were included based on the inclusion criteria. After excluding 47 patients based on the exclusion criteria, 741 were finally included (**Figure 1**). The baseline clinical characteristics are presented in **Table 1**. Of the 741 patients, 255 (34.4%) had poor outcome (mRS >2), and 43 (5.8%) died.

**Table 1:** Baseline characteristics of patients according to presence/absence of poor outcome and death.

	Total	Favorableoutcome (mRS ≤ 2)	Poor outcome (mRS > 2)	*P*	Alive	Dead	*P*
N, n (%)	741	486 (65.6)	255 (34.4)		698 (94.2)	43 (5.8)	
Age, years, median (IQR)	62 (53-69)	60 (52-68)	64 (55-71)	0.001	61 (53-68)	68 (58-73)	0.002
Females, n (%)	202 (27.3)	131 (27.0)	71 (27.8)	0.796	189 (27.1)	13 (30.2)	0.652
Smoking, n (%)	408 (55.1)	280 (57.6)	128 (50.2)	0.054	382 (54.7)	26 (60.5)	0.463
Alcohol consumption, n (%)	320 (43.2)	209 (43.0)	111 (43.5)	0.891	301 (43.1)	19 (44.2)	0.891
Hypertension, n (%)	387 (52.2)	241 (49.6)	146 (57.3)	0.047	366 (52.4)	21 (48.8)	0.647
Diabetes, n (%)	210 (28.3)	131 (27.0)	79 (31.0)	0.248	190 (27.2)	20 (46.5)	0.006
Coronary artery disease, n (%)	138 (18.6)	87 (17.9)	51 (20.0)	0.486	131 (18.8)	7 (16.3)	0.684
Atrial fibrillation, n (%)	43 (5.8)	25 (5.1)	18 (7.1)	0.290	38 (5.4)	5 (11.6)	0.092
Previous stroke, n (%)	107 (14.4)	64 (13.2)	43 (16.9)	0.174	101 (14.5)	6 (14.0)	0.926
Antihypertensive drugs, n (%)	288 (38.9)	176 (36.2)	112 (43.9)	0.041	274 (39.3)	14 (32.6)	0.382
Hypoglycemic agents, n (%)	123 (16.6)	79 (16.3)	44 (17.3)	0.728	113 (16.2)	10 (23.3)	0.227
Antiplatelet agents, n (%)	94 (12.7)	58 (11.9)	36 (14.1)	0.396	85 (12.2)	9 (20.9)	0.094
SBP, mmHg, median (IQR)	154 (139-166)	152 (138-164)	156 (142-169)	0.005	154 (139-165)	156 (134-172)	0.788
DBP, mmHg, median (IQR)	90 (81-98)	90 (80-98)	89 (82-98)	0.674	90 (81-98)	88 (79-98)	0.751
Blood glucose, mmol/L, median (IQR)	7.08 (6.24-8.82)	6.99 (6.18-8.86)	7.28 (6.33-8.80)	0.170	7.08 (6.23-8.80)	7.06 (6.33-9.56)	0.648
Time to treatment, min, median (IQR)	180 (142-231)	182 (142-230)	180 (139-231)	0.671	180 (141-230)	181 (162-237)	0.298
Baseline NIHSS score, median (IQR)	8 (5-12)	7 (4-11)	11 (7-14)	<0.001	8 (5-12)	13 (10-16)	<0.001
Anterior circulation, n (%)	583 (78.7)	374 (77.0)	209 (82.0)	0.114	552 (79.1)	31 (72.1)	0.277
**TOAST**				<0.001			0.034
Large-artery atherosclerosis, n (%)	240 (32.4)	120 (24.7)	120 (47.1)		221 (31.7)	19 (44.2)	
Small-vessel occlusion, n (%)	349 (47.1)	263 (54.1)	86 (33.7)		337 (48.3)	12 (27.9)	
The other types, n (%)	152 (20.5)	103 (21.2)	49 (19.2)		140 (20.1)	12 (27.9)	
**On admission**							
Platelet count, 10^9/L, median (IQR)	200 (169-233)	198 (170-230)	203 (168-235)	0.462	200 (170-234)	200 (168-220)	0.610
Lymphocyte count, 10^9/L, median (IQR)	1.70 (1.25-2.23)	1.71 (1.25-2.23)	1.66 (1.24-2.24)	0.607	1.70 (1.24-2.24)	1.57 (1.35-2.14)	0.897
PLR, median (IQR)	118.6 (90.0-155.3)	118.8 (88.2-154.7)	118.6 (91.3-156.6)	0.473	118.6 (90.2-155.5)	124.2 (84.4-148.5)	0.971
**24 h after rtPA**							
Platelet count, 10^9/L, median (IQR)	205 (171-237)	204 (170-237)	206 (173-239)	0.295	205 (170-238)	199 (179-219)	0.519
Lymphocyte count, 10^9/L, median (IQR)	1.76 (1.32-2.15)	1.84 (1.41-2.23)	1.58 (1.18-2.03)	<0.001	1.78 (1.35-2.16)	1.32 (0.90-1.85)	<0.001
PLR, median (IQR)	116.6 (91.8-151.5)	109.2 (87.4-141.3)	131.4 (104.1-173.7)	<0.001	115.3 (91.2-149.5)	146.0 (112.9-209.7)	<0.001

Continuous and categorical variables were compared using the Mann–Whitney U-test and chi-squared test. mRS, Modified Rankin Scale; IQR, interquartile range; SBP, systolic blood pressure; DBP, diastolic blood pressure; NIHSS, National Institutes of Health Stroke Scale; TOAST, the Trial of Org 10172 in Acute Stroke Treatment; PLR, platelet-to-lymphocyte ratio; rtPA, recombinant tissue plasminogen activator.

Patients with poor outcome tended to be older and more likely to have hypertension, to be on antihypertensive therapy, have large-artery atherosclerosis, and have higher SBP and NIHSS scores (all *P*<0.05). Patients who died within 3 months were older and more likely to have diabetes, higher NIHSS scores, and a higher proportion of large-artery atherosclerosis (all *P*<0.05) (**Table 1**).

### Changes in the PLR before and after rtPA

**Figure 2:**
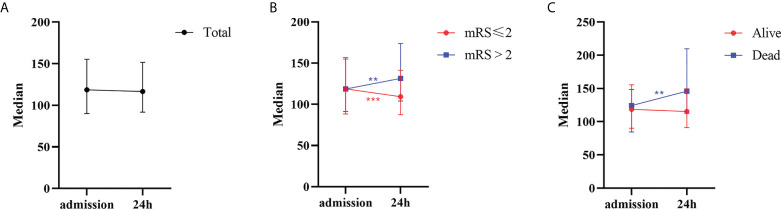
The 24 h dynamic change of PLR after rtPA in patients. **(A)** Patients in total. **(B)** Patients with favorable (mRS ≤ 2) or poor (mRS>2) outcome. **(C)** Patients with alive or dead status. PLR, platelet-to-lymphocyte ratio; rtPA, recombinant tissue plasminogen activator; mRS, Modified Rankin Scale. **P<0.01; ***P<0.001.

Overall, no significant difference was noted between the PLR on admission and that 24 h after rtPA (*P*=0.462). In the poor-outcome (mRS >2) group, the PLR increased significantly 24 h after rtPA treatment (median: 118.6 vs. 131.4, *P*=0.004). In contrast, the PLR decreased significantly 24 h after rtPA treatment compared with the value before rtPA therapy (median: 118.8 vs. 109.2, *P*<0.001) in the favorable-outcome group. In the deceased group, the PLR also increased significantly 24 h after rtPA treatment (median: 124.2 vs. 146.0, *P*=0.002) (**Supplemental Table 1** and **Figure 2**).

### Association between the PLR and outcomes

The 24 h PLR after rtPA therapy was significantly higher in patients with poor outcome (median: 109.2 vs. 131.4, *P*<0.001) and death (median: 115.3 vs. 146.0, *P*<0.001) than in those without (**Table 1**). Multivariate analysis revealed that higher PLR 24 h after rtPA was independently associated with an increased risk of poor outcome (OR=1.005; 95% CI: 1.002–1.008; *P*<0.001) and death (OR=1.008; 95% CI: 1.003–1.012; *P*=0.001). However, the PLR on admission was not associated with an increased risk of poor outcome (OR=1.001; 95% CI: 0.998–1.003; *P*=0.534) or death (OR=0.998; 95% CI: 0.992–1.003; *P*=0.421) (**Table 2**).

**Table 2:** Univariate and multivariable logistic regression analysis of PLR associated with poor outcome and death after rtPA.

		OR	95% confidence interval		*P*
			Lower bound	Upper bound	
PLR on admission (per 1 unit)					
Poor outcome	Model 1^a^	1.001	0.999	1.004	0.192
	Model 2^b^	1.001	0.998	1.003	0.607
	Model 3^c^	1.001	0.998	1.003	0.534
Death	Model 1^a^	1.000	0.995	1.004	0.856
	Model 2^b^	0.999	0.994	1.003	0.579
	Model 3^c^	0.998	0.992	1.003	0.421
PLR at 24 h after rtPA (per 1 unit)					
Poor outcome	Model 1^a^	1.007	1.004	1.010	<0.001
	Model 2^b^	1.005	1.002	1.008	<0.001
	Model 3^c^	1.005	1.002	1.008	<0.001
Death	Model 1^a^	1.009	1.005	1.013	<0.001
	Model 2^b^	1.007	1.003	1.011	0.002
	Model 3^c^	1.008	1.003	1.012	0.001

a unadjusted.

b adjusted for age, diabetes, baseline NIHSS score, TOAST.

c Adjusting for age, sex, smoking, alcohol drinking, hypertension, diabetes, coronary artery disease, atrial fibrillation, previous stroke, antihypertensive drugs, hypoglycemic agents, antiplatelet agents, systolic blood pressure, diastolic blood pressure, blood glucose, time to treat, baseline NIHSS score, anterior circulation and TOAST.

PLR, platelet-to-lymphocyte ratio; rtPA, recombinant tissue plasminogen activator, NIHSS, National Institutes of Health Stroke Scale, TOAST, the Trial of Org 10172 in Acute Stroke Treatment.

### Individualized prediction model

**Figure 3:**
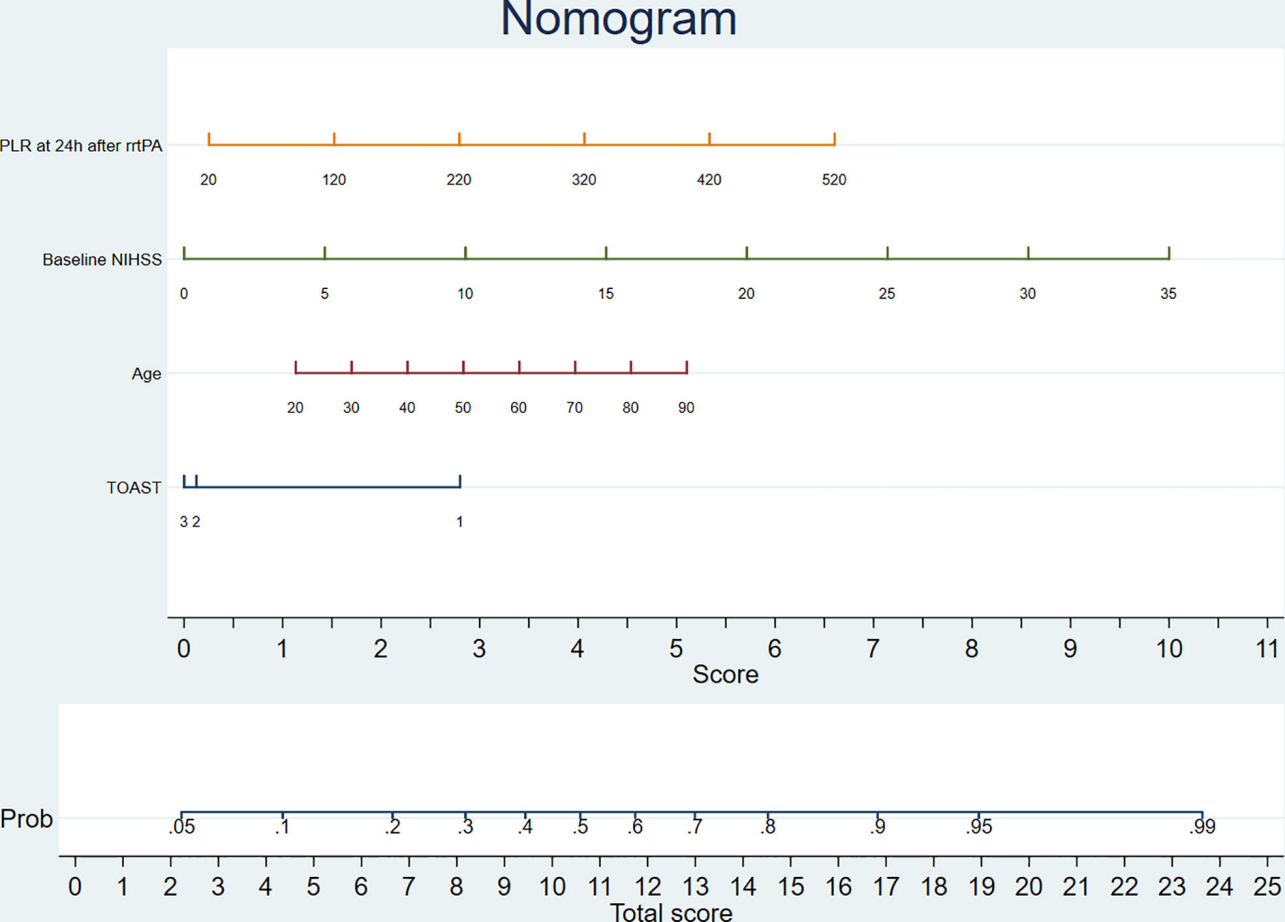
The nomogram for predicting poor outcome. TOAST: 1, large-artery atherosclerosis; 2, small-vessel occlusion; 3, the other types. PLR, platelet-to-lymphocyte ratio; rtPA, recombinant tissue plasminogen activator; NIHSS, National Institutes of Health Stroke Scale; TOAST, the Trial of Org 10172 in Acute Stroke Treatment.

**Figure 4:**
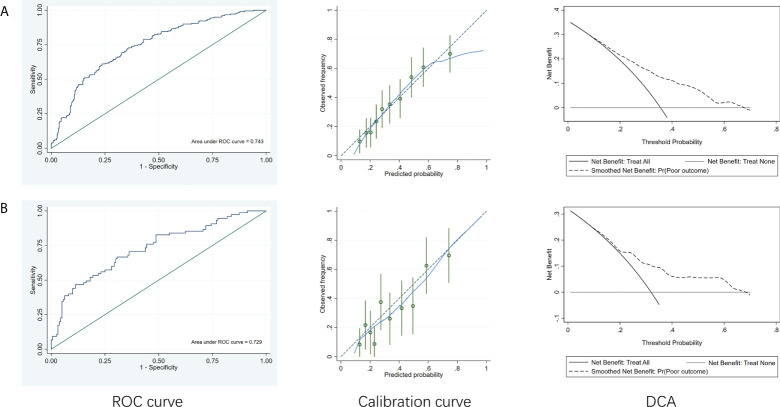
ROC curves, calibration curve and DCA of the model to predict poor outcome. **(A)** Training group. **(B)** Validation group. ROC, receiver operating characteristic curve; DCA, decision curve analysis.

**Figure 5:**
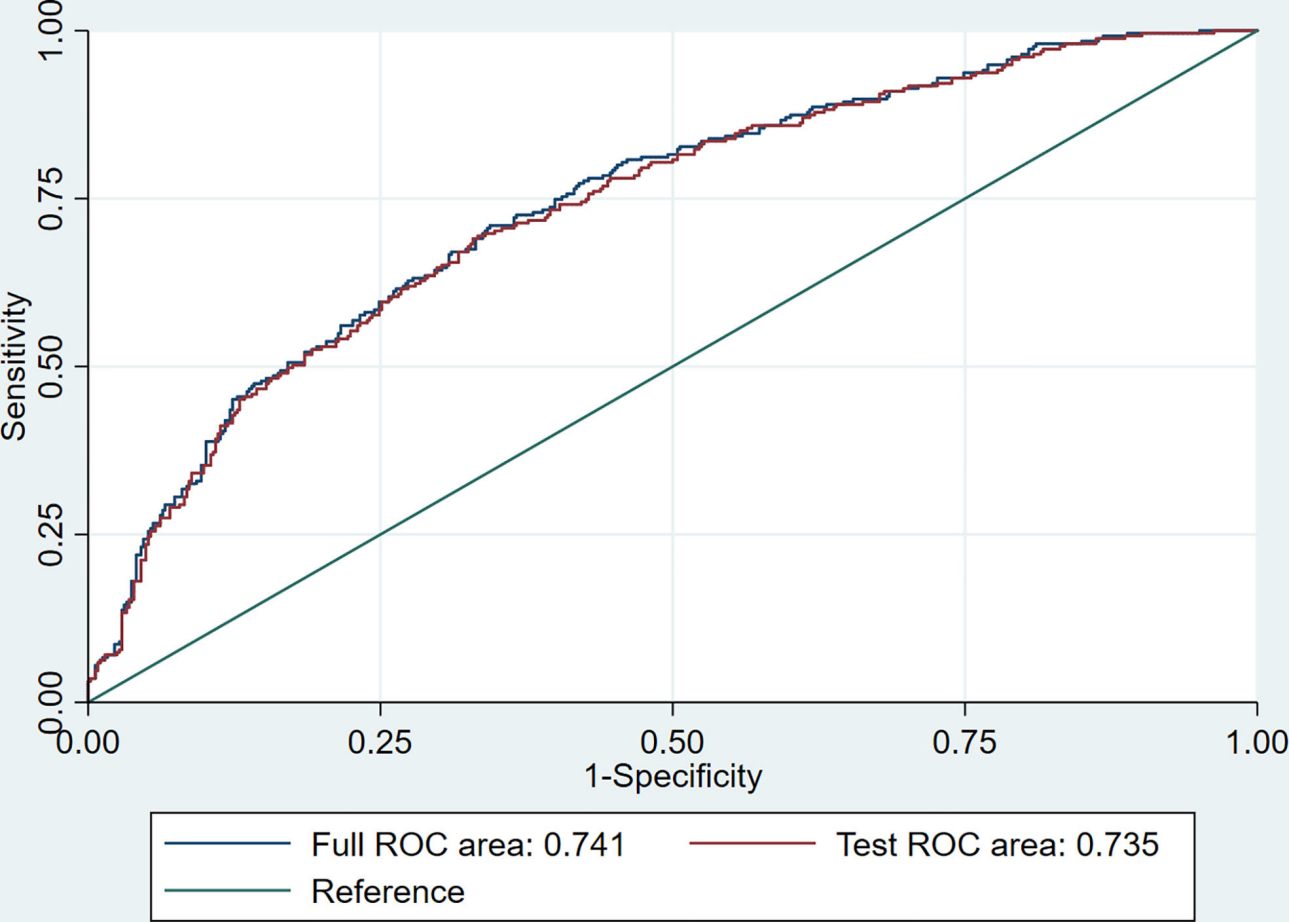
The ROC curve for the 10-fold cross-validation.

A comparison of the training and validation groups is shown in **Supplemental Table 2**. The individualized prediction model for poor outcome comprised age (continuous variables), baseline NIHSS score (continuous variables), TOAST subtype (categorical variables), and the 24-h PLR after rtPA (continuous variables), based on the results of the multivariate logistic regression analysis. The nomogram is shown in **Figure 3**. The AUC-ROC values of the training and validation groups were 0.743 (95% CI: 0.692–0.787) and 0.729 (95% CI: 0.659–0.800) respectively, indicating favorable discrimination. The cutoff point for the training and validation group was 0.393 with a sensitivity of 61%, a specificity of 76%, and 0.348 with a sensitivity of 67%, a specificity of 69%, respectively. The calibration curve indicated a favorable predictive accuracy in both the training and validation groups. The Hosmer–Lemeshow test yielded *P*=0.977 and *P*=0.571 for the training and validation groups, respectively, indicating favorable calibration. DCA results were similar in the training and validation groups, reflecting a relatively favorable clinical net benefit (**Figure 4**). The ROC curve for the 10-fold cross-validation is given in **Figure 5**.

## Discussion

This study attempted to explore the changes in the PLR before and 24 h after rtPA as well as evaluate their relationship with outcomes in acute ischemic stroke patients. We found the PLR increased 24 h after rtPA treatment in patients with poor outcome and death. This result indicated that instead of the PLR on admission, the 24h-PLR after rtPA was an independent risk factor for poor outcome and death. In addition, the individualized prediction model for poor outcome, which comprised age, baseline NIHSS score, TOAST subtype, and the 24-h PLR after rtPA, indicated favorable discrimination, calibration, and clinical usefulness. Our results suggest that the 24-h PLR after rtPA has prognostic value for poor outcome in patients who have undergone intravenous thrombolysis; thus, the PLR could be considered a possible target for interventions that improve outcomes in these patients.

Previous studies have shown that the PLR may be a strong biomarker used for the prognosis of acute ischemic stroke. For example, a recent study suggested that the PLR is potentially useful as an independent predictor of functional outcome at 3 months in patients with acute ischemic stroke (15). Simultaneously, a study by Altintas et al. (16) conducted in acute ischemic stroke patients undergoing endovascular therapy found the correlation between lower PLR values and a better clinical outcome (mRS ≤2). However, to the best of our knowledge, only two studies have explored the association between the PLR and outcomes in patients who have undergone intravenous thrombolysis. Xu et al. (12) collected lymphocyte- and platelet-count data within 24 h and found a higher PLR to be independently associated with poor outcome and death at 3 months in patients with stroke treated with intravenous thrombolysis. In another observational study of patients undergoing rtPA treatment, researchers found the PLR on admission before intravenous thrombolysis to be associated with early neurological improvement and deterioration (11). No study has investigated the predictive value of the PLR before intravenous thrombolysis for long-term clinical outcomes. Moreover, these previous studies exclusively reported a single time point for the PLR, and the time point at which blood samples were collected for PLR calculation was heterogeneous. Conversely, our study collected platelet- and lymphocyte-count data both on admission and 24 h after thrombolysis and found that only the 24-h PLR after rtPA was independently associated with poor outcome and death, while the PLR on admission showed no association with these outcomes.

In addition, we investigated the 24-h changes in the PLR after rtPA. Various components of the immune system change dynamically after stroke and may potentially have detrimental or beneficial effects depending on the stage of the development of ischemia. Therefore, understanding PLR changes during stroke and determining the time point at which the PLR is effective as a predictor of patient prognosis are of paramount importance (17, 18). Our study found the PLR to increase 24 h after rtPA treatment in patients with poor outcome and death; nonetheless, the trends in patients with favorable outcome were completely contrary. This mechanism may be due to the following reasons. Lymphocytes exhibit significant temporal variation after ischemic stroke (19, 20), and they accumulate in cerebral vessels at a later time within 24 h of the onset of stroke (21). A decreased lymphocyte count after ischemic stroke potentially aggravates the injury to neurons, and further exacerbate cerebral infarction and neurological deficits (22). Some subtypes of lymphocytes, such as regulatory T cells (Tregs), are known to attenuate inflammation and may exhibit brain protective qualities by producing anti-inflammatory cytokines in the process of acute stroke (19, 23). The consumption of Tregs after stroke profoundly exacerbates functional outcome (24). Furthermore, the decrease of lymphocyte count can also reflect the activation of the renin–angiotensin system, which further increases the production of proinflammatory cytokines, promoting ischemic injury (22, 25–27). In addition, lymphocytopenia may also increase the risk of infection in patients with stroke, which is associated with poor outcome (28). In contrast, studies have demonstrated that excessive activation and accumulation of platelets may reflect a greater tendency toward the inflammatory response and thrombosis, leading to hampered stroke recovery and poor prognosis (29, 30). Moreover, the mechanisms by which the prognostic value of the 24-h PLR after rtPA appears to be greater than that at baseline have not been well established. An increase in platelet count after thrombolytic therapy may be responsible for delayed thrombosis, leading to reocclusion and rethrombosis (31). A previous study revealed that rtPA potentially induces the elevation of matrix metalloproteinase-9 and chemokine ligand-2, which mediate blood–brain barrier disruption and may lead to hemorrhagic transformation. During this period, Tregs potentially eliminate the excess of these two inflammatory factors, thus decreasing the risk of brain damage and poor prognosis (24). Therefore, we speculated that the PLR at baseline may not be used as an ideal indicator to reflect the patient’s condition dynamically and comprehensively, whereas the post rtPA ratio would be a more valuable indicator.

The PLR, as an index, combines platelets and lymphocytes, which results in the unique advantage of linking key pathways both in thrombus formation and the inflammation process (32). The PLR index has potential superiority to an absolute platelet or lymphocyte count in the prediction of acute ischemic stroke prognosis. Firstly, the PLR combines two predictors which represent two inverse immunologic pathways (33). Secondly, individual platelet and lymphocyte count are not as stable as a combined ratio like PLR, as these values are fragile and potentially altered by several physiologic and pathologic conditions (34–36). Therefore, the PLR shows its reliability and rationality in predicting the risk of poor outcome and death for acute ischemic stroke patients.

In addition, we established an individualized prediction model for predicting poor outcome, which comprised age, baseline NIHSS score, TOAST subtype, and the 24-h PLR after rtPA. The AUC-ROC, Hosmer–Lemeshow test, and DCA results in the training and validation groups were similar, indicating that our model had favorable stability. Moreover, the results of the AUC-ROC, Hosmer–Lemeshow tests, and DCA suggested that our model exhibited favorable discrimination, calibration, and clinical usefulness. Thus, we considered our model based on the 24-h PLR after rtPA to have favorable prognostic value for poor outcome in patients undergoing rtPA treatment.

Notwithstanding, our study had certain limitations. First, this paper is a retrospective analysis of data from single-center hospitals. Second, we examined the PLR only at baseline and 24h after thrombolysis, where the two time points cannot fully reflect a dynamic change in the PLR throughout the entire process of stroke. In addition, the definite mechanisms for this have not been fully elucidated. Further investigation is required to explore the relevant mechanisms underlying the findings of this study.

## Conclusion

In conclusion, we found the PLR to be a variable that is associated with the risks of poor outcome and death in patients with acute ischemic stroke 24 h after rtPA thrombolysis, rather than before. Furthermore, PLR is potentially useful as a simple, novel, and inexpensive method of predicting patient prognosis.

## Data availability statement

The raw data supporting the conclusions of this article will be made available by the authors, without undue reservation.

## Ethics statement

The studies involving human participants were reviewed and approved by the Ethics Committee of the First Hospital of Jilin University (2016-294). The patients/participants provided their written informed consent to participate in this study.

## Author contributions

YY and Z-NG were responsible for study design. Material preparation, data collection and analysis were performed by Y-YS, M-QW and YW. The first draft of the manuscript was written by Y-YS. XS, YQ, H-JZ, S-JW, X-LY, HJ and PZ helped revise the manuscript. All authors read and approved the final manuscript.

## Funding

This work was supported by the National Natural Science Foundation of China (Grant No. 81901192) to XS, the Science and technology department of jilin province (20180623052TC), and the Jilin Provincial Key Laboratory (20190901005JC) to YY.

## Acknowledgments

The authors thank the patients and their families and appreciate the study participants for their assistance in this study.

## Conflict of interest

The authors declare that the research was conducted in the absence of any commercial or financial relationships that could be construed as a potential conflict of interest.

## Publisher’s note

All claims expressed in this article are solely those of the authors and do not necessarily represent those of their affiliated organizations, or those of the publisher, the editors and the reviewers. Any product that may be evaluated in this article, or claim that may be made by its manufacturer, is not guaranteed or endorsed by the publisher.
