# Supplementary material for: Platelet-to-lymphocyte ratio at 24h after thrombolysis is a prognostic marker in acute ischemic stroke patients
Source: Front Immunol. 2022 Sep 26;13:1000626. doi: 10.3389/fimmu.2022.1000626 (PMC9549955; doi:10.3389/fimmu.2022.1000626)
Supplement: Supplementary file 1 [file Table_1.docx]

**Supplemental Table** **1.** The 24 h change PLR after rtPA.

|  | On admission (0h) | 24 h after rtPA | Z | *P* |
| --- | --- | --- | --- | --- |
| Total | 118.6(90.0-155.3) | 116.6(91.8-151.5) | -0.735 | 0.462 |
| Favorable outcome (mRS≤2) | 118.8(88.2-154.7) | 109.2(87.4-141.3) | -3.489 | <0.001 |
| Poor outcome (mRS>2) | 118.6(91.3-156.6) | 131.4(104.1-173.7) | -2.919 | 0.004 |
| Alive | 118.6(90.2-155.5) | 115.3(91.2-149.5) | -1.718 | 0.086 |
| Dead | 124.2(84.4-148.5) | 146.0(112.9-209.7) | -3.091 | 0.002 |

The paired Wilcoxon signed-rank test was performed to compare the PLR before and 24 h after rtPA. PLR, platelet-to-lymphocyte ratio; rtPA, recombinant tissue plasminogen activator; mRS, Modified Rankin Scale.

**Supplemental Table 2.** Comparison of the training and validation groups.

|  | Training (n=506) | Validation (n=235) | *P* |
| --- | --- | --- | --- |
| Age, years, median (IQR) | 61(53-68) | 62(53-70) | 0.377 |
| Females, n (%) | 139(27.5) | 63(26.8) | 0.851 |
| Smoking, n (%) | 282(55.7) | 126(53.6) | 0.590 |
| Alcohol consumption, n (%) | 224(44.3) | 96(40.9) | 0.382 |
| Hypertension, n (%) | 266(52.6) | 121(51.5) | 0.784 |
| Diabetes, n (%) | 134(26.5) | 76(32.3) | 0.100 |
| Coronary artery disease, n (%) | 94(18.6) | 44(18.7) | 0.962 |
| Atrial fibrillation, n (%) | 30(5.9) | 13(5.5) | 0.830 |
| Previous stroke, n (%) | 73(14.4) | 34(14.5) | 0.988 |
| Antihypertensive drugs, n (%) | 199(39.3) | 89(37.9) | 0.705 |
| Hypoglycemic agents, n (%) | 80(15.8) | 43(18.3) | 0.397 |
| Antiplatelet agents, n (%) | 63(12.5) | 31(13.2) | 0.778 |
| SBP, mmHg, median (IQR) | 154(140-165) | 154(137-166) | 0.504 |
| DBP, mmHg, median (IQR) | 89(81-97) | 90(81-98) | 0.550 |
| Blood glucose, mmol/L, median (IQR) | 7.20(6.26-8.72) | 6.88(6.20-9.24) | 0.691 |
| Time to treatment, min, median (IQR) | 180(137-230) | 183(150-233) | 0.404 |
| Baseline NIHSS score, median (IQR) | 9(5-12) | 8(5-12) | 0.324 |
| Anterior circulation, n (%) | 401(79.2) | 182(77.4) | 0.577 |
| **TOAST** |  |  | 0.012 |
| Large-artery atherosclerosis, n (%) | 156(30.8) | 84(35.7) |  |
| Small-vessel occlusion, n (%) | 231(45.7) | 118(50.2) |  |
| The other types, n (%) | 119(23.5) | 33(14.0) |  |
| PLR at 24 h after rtPA, median (IQR) | 118.1(93.6-151.8) | 111.2(88.4-150.9) | 0.296 |

Continuous and categorical variables were compared using the Mann–Whitney U-test and chi-squared test. IQR, interquartile range; SBP, systolic blood pressure; DBP, diastolic blood pressure; NIHSS, National Institutes of Health Stroke Scale; TOAST, the Trial of Org 10172 in Acute Stroke Treatment; PLR, platelet-to-lymphocyte ratio; rtPA, recombinant tissue plasminogen activator.
